# Symmetry-breaking induced magnetic Fano resonances in densely packed arrays of symmetric nanotrimers

**DOI:** 10.1038/s41598-019-39779-x

**Published:** 2019-02-27

**Authors:** Ning Wang, Matthias Zeisberger, Uwe Huebner, Vincenzo Giannini, Markus A. Schmidt

**Affiliations:** 1 Leibniz Institute of Photonic Technology, Albert-Einstein-Str. 9, 07745 Jena, Germany; 2 The Blackett Laboratory, Department of Physics, Imperial College London, London, SW7 2AZ UK; 3 Instituto de Estructura de la Materia (IEM-CSIC), Consejo Superior de Investigaciones Científicas, Madrid, 28006 Spain; 4 Abbe School of Photonics and Faculty of Physics, Max-Wien-Platz 1, 07743 Jena, Germany; 5 Otto Schott Institute of Materials Research, Fraunhoferstr. 6, 07743 Jena, Germany

## Abstract

Due to unique properties and great design flexibilities, Fano resonances represent one of the most promising optical features mediated by metallic nanostructures, while the excitation of some Fano modes is impossible due to symmetry reasons. The aim of this work is to show that dense lattice arrangements can have a profound impact on the optical properties of nanostructures and, in particular, can enable the excitation of otherwise dark modes. Here, we demonstrate this concept using the example of rectangular arrays of symmetric trimers packed so densely that the coupling between neighbouring unit cells imposes a symmetry break, enabling the excitation of magnetic Fano resonances. We found that in experiments as well as in simulations, electric and magnetic Fano resonances can be simultaneously formed in cases where the inter-trimer distances are sufficiently small. By analysing the transition from an isolated trimer mode into a regime of strong near-field coupling, we show that by modifying the rectangular unit cell lengths due to the symmetry mismatch between lattice and trimer, two types of Fano resonances can be found, especially magnetic Fano resonances with loop-type magnetic field distributions within the centre of each trimer, which can be either enhanced or suppressed. In addition, the influence of the refractive index environment was measured, showing sensitivity values of approximately 300 nm/RIU. Our work provides fundamental insights into the interaction of the lattice and nanostructure response and paves the way towards the observation of novel optical excitations.

## Introduction

In recent years, nano-optics has attracted substantial attention, with a large number of studies^1–3^ carried out to understand the interaction between light and matter on the nanoscale. By using well-designed plasmonic nanoantennae^4,5^, sophisticated photonic functionalities have been realized on ultrasmall geometric footprints on the basis of, e.g., deep sub-wavelength metasurfaces^6,7^, hybrid plasmonic nanosensors^8^, and monolithic fibre nanoprobes^9^.

One particular striking effect mediated by nanostructures is the Fano resonance, which arises from interference between a broadband bright mode and a narrowband dark mode^10,11^. This modal interaction dramatically changes the lineshape of scattered light by introducing a narrow dip into the extinction spectrum. Due to the low damping of the dark mode, the electromagnetic near-field at the wavelength of the Fano resonance is greatly enhanced, which has the consequence that the scattered light is enhanced accordingly^12^. To achieve such a unique effect, numerous types of nanostructures such as single split nanodots^12,13^, nanocavities^14^, nanosphere dimers^15^, dolmen-like nanostructures^14,16^, plasmonic clusters^17^ and oligomers^18–20^ are currently being employed. Among these, nanotrimers consisting of three close-packed nanoparticles represent a promising design for realizing the Fano resonance (details of the hybridization are discussed in the Supplementary Information sec. 6). As first experimentally demonstrated in 2010 for the example of self-assembled metal-dielectric spheres^21^, trimers can exhibit a strong magnetic resonance. Later, several studies^22–25^ tried to obtain this magnetic enhancement in trimers by symmetry breaking within the trimer and have successfully shown excitation of magnetic-based Fano resonances with the magnetic resonance being the dark mode. In contrast to an internal symmetry break, the aim of this study is to show that excitation of magnetic Fano resonances in symmetric trimers can be achieved by placing them into densely packed arrays, with the symmetry breaking induced by the lattice arrangement. Here, the trimers are arranged in a rectangular lattice (two-fold symmetry) breaking the three-fold symmetry of the otherwise symmetric trimer unit.

For a majority of the Fano resonances mentioned above, arrays of trimer arrays are used where inter-trimer distances are chosen such to significantly exceed the individual trimer resonance wavelength^16^. In a denser configuration, the compact geometrical arrangement leads to strong near-field unit cell interactions, with the diffractive coupling of localized plasmonic resonances^26,27^ further modifying the resonance lineshape. For instance, Chen Yan *et al*.^16^ have demonstrated that the optical response of arrays of nanodolmens can be significantly altered by the near-field coupling to adjacent unit cells. By varying the periodicities, the far-field response of the plasmonic system can be evolved into a tight-binding regime or a hybridization regime showing a combined response of the individual trimer response and of the diffraction of the array. However, a detailed investigation for this type of hybrid response between nanostructures and inter-unit cell diffractive coupling has not been intensively carried out to date.

In this report, we reveal the optical properties of densely packed arrays of nanotrimers and their dependence on the geometrical parameters, such as intra-trimer gaps and array pitch. We conducted a set of experiments as well as numerical calculations with a focus on the trimer modes and found that magnetic Fano resonances can be excited for the symmetric trimer geometry when the trimers are arranged in densely packed arrays, with the symmetry breaking induced by the lattice arrangement and not by the individual trimer unit. The report is arranged as follows: First, we analyse the extinction spectrum of the trimer array and find a magnetic Fano resonance mode, identified by its experimental spectral features and simulated near-field distributions^28,29^. Second, to further explore the impact of the near-field coupling effect on Fano resonances, extinction spectra with varying trimer-to-trimer distances were modelled and analysed. Finally, the trimer arrays were optically characterized under various refractive index (RI) environments. The results indicate that an asymmetric refractive index arrangement between substrate and superstrate weakens the formation of magnetic Fano resonances.

## Design and Fabrication

**Figure 1:**
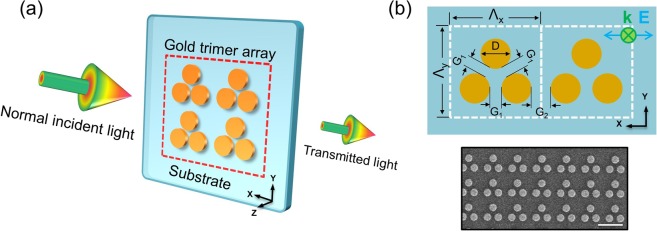
Schematic of the square lattice trimer array incl. the relevant structural parameters. (**a**) Sketch of four trimers located on a silica substrate and excited by polarization-controlled light. Each trimer unit is composed of three symmetrically arranged gold nanodisks. The red frame highlights the dense distribution of the trimer array. (**b**) Details of the symmetric trimer geometry (top) with a corresponding scanning electron micrograph (SEM) image (bottom). The symmetric trimer shares the same inter-dot distances G_1_ between all three nanodisks. Here, G_1_ and G_2_ are named as the intra-trimer gap and inter-trimer gap, respectively. The blue double-headed arrow with the green cross indicates normal incidence and the light polarization along the x-axis. The scale bar in the SEM image refers to 1 μm.

The trimer array is fabricated by lift-off technology using electron beam lithography and dry etching^30^ (a sketch of one sample is shown in Fig. 1). The fabrication details can be found in the Supplementary Information (SI) in Sec. 3. The array of trimers has a size of 110 × 110 μm^2^ covering the beam spot of the 20× objectives used in the optical characterization experiments. Each trimer unit is composed of three identical planar gold nanodisks (diameter 425 nm) with a G_1_ (intra-trimer gap, sketch in Fig. 1(b)) of 75 nm. The choice of a gold film thickness of 40 nm was mostly based on preliminary fabrication experiments (i.e., this thickness gave the best nanostructures) and has no substantial influence on the mode formation discussed in the remaining part of the manuscript. For the specific sample used in the characterization and RI sensing experiments, the value for G_2_ (inter-trimer gap, sketch in Fig. 1(b)) was chosen to be 175 nm, corresponding to an array pitch Λ of 1100 nm. The scanning electron micrograph (SEM) image shown in Fig. 1(b) clearly demonstrates the compactness of the trimer array. Note that in all the extinction data, the polarization of the incident light is along the x-axis.

## Discussions

### Magnetic Fano resonance in closely packed trimer arrays

**Figure 2:**
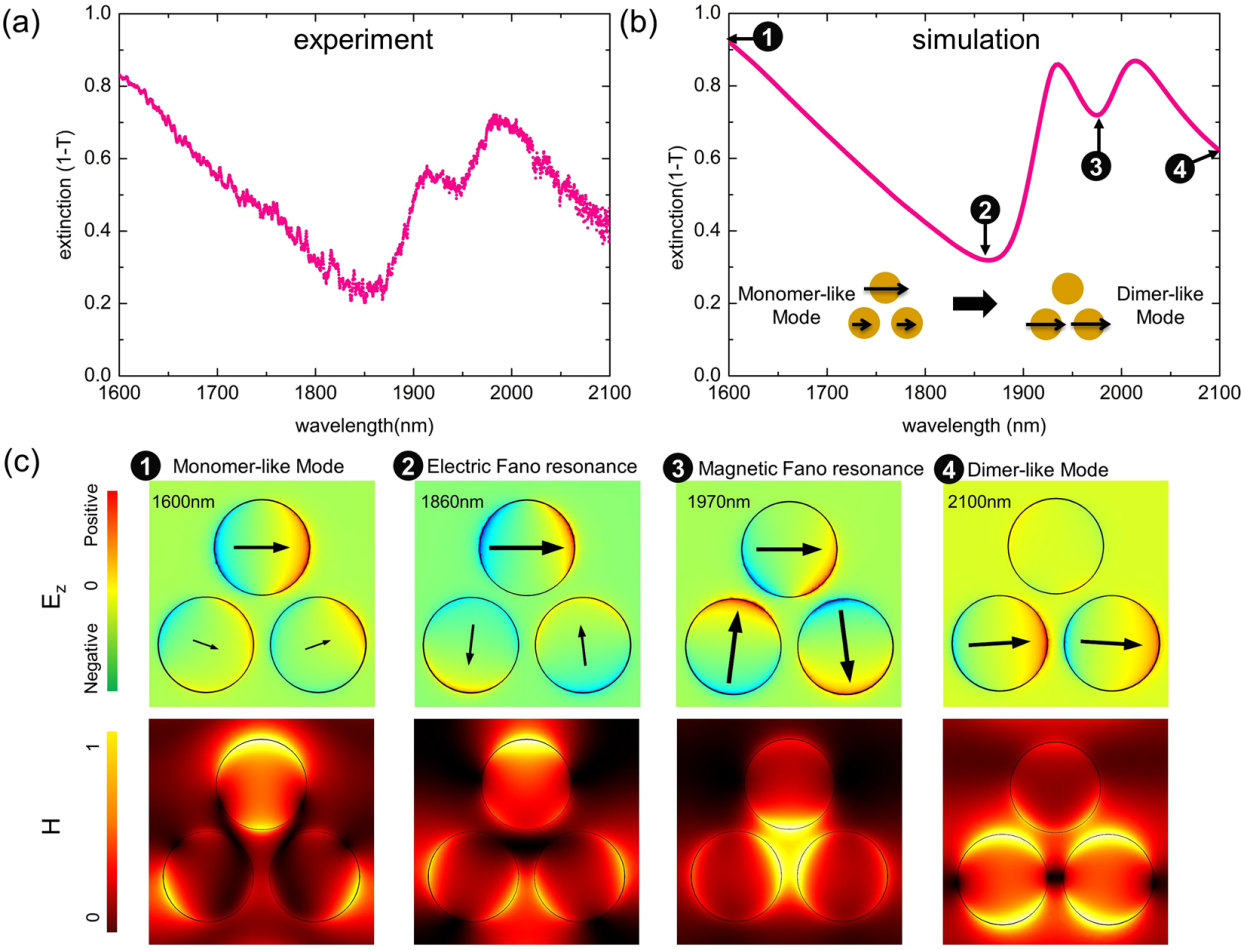
(**a**) Measured extinction spectrum and (**b**) corresponding numerical simulation for a trimer array with selected near-field patterns shown in (**c**) (array pitch Λ: 1100 nm, SEM in Fig. 1(b)). Polarization directions (arrows) and distribution of the out-of-plane electric field component E_*z*_ (colour indicates the charge distribution) of one trimer unit cell at a fixed point of time are shown in the upper row of (**c**) together with the normalized magnetic field distributions in the lower row. The number labels correspond to the wavelengths highlighted in (**b**). Each image shown in (**c**) corresponds to an area of 1 × 1 μm^2^.

As a first step, we characterize the optical response of the trimer array (D: 425 nm H: 40 nm G_1_: 75 nm) for a fixed pitch (1100 nm, G_2_: 175 nm). In Fig. 2, we show the measured and simulated trimer extinction spectra (from 1600 nm to 2100 nm) together with the simulated near-field patterns at selected spectral positions. Within this specific spectral domain, each individual nanoparticle of one trimer behaves like a dipole, meaning that a more complex (multipolar) response for the individual nanodot does not need to be considered. According to the spectra shown in Figs S1 and S2 (Supplementary Information section 1 and 2), the spectral response of closely packed trimer arrays exhibits a highly asymmetrical lineshape and a strong polarization dependence, which contrasts with the isolated trimers discussed in refs.^31–34^. Hence, we carried out a set of studies with a focus on these densely packed trimers. In addition, only x-polarized incident light is applied since the polarization influence is beyond the scope of this work.

To obtain a homogeneous RI environment along the vertical direction (Fig. 2(a)), the trimer samples are immersed in a liquid that matches the RI of the substrate (n = 1.44). For spectral measurements, a commercial broadband supercontinuum source (NKT Photonics SuperK Compact) was employed for illumination, with the transmitted light recorded by a spectrometer (Instrument Systems SP320-124). Two transmitted spectra were recorded with one beam directed to the respective trimer array and the other passing through an unstructured part of the silica substrate. The experimental transmission T is then obtained by the power ratio of the two spectra. Please note that in this report, we present extinction spectra obtained using (1 - T).

Corresponding numerical calculations (Fig. 2(b)) based on the finite-element-method (FEM) are also conducted in the index-matched configuration by applying periodic boundary conditions. The simulated electric field (E_*z*_, i.e., the component perpendicular to the sample surface - see Fig. 2(c) upper row) plus polarization arrows indicate the charge distributions for the trimer. The electric field data are taken from a plane that is located 1 nm above the disks (z = 41 nm), with the polarization distribution calculated within the middle plane (z = 20 nm) of the nanodisks. In addition, the spatial distribution of the magnetic field (|H| at z = 50 nm) is presented to help one to understand the formed trimer modes in this lattice arrangement. The experimental spectrum coincides well with numerical simulations (Fig. 2(a,b)) where the two main minima are both fully captured (one minimum is near to 1850 nm, and the other minimum is close to 1950 nm) even though the amplitude of the features are slightly different.

At the sides of the investigated spectral domain (points 1 and 4 in Fig. 2(b)), the related field patterns (Fig. 2(c)) exhibit dipolar excitations of the upper particle (monomer excitation) and the two lower particles (dimer excitation), showing a transition from a monomer-like mode to a dimer-like mode (schematically illustrated in Fig. 2(b)). Between these two cases, two extinction minima (points 2 and 3 in Fig. 2) are found corresponding to different Fano resonance modes. At a wavelength of 1860 nm (point 2), a dipolar excitation of the upper and quadrupolar excitations of the two lower dots are observed, resembling a dolmen-like electric Fano pattern^14,16^.

It is of great interest to note the loop-like polarization distribution (named as magnetic Fano resonance in Fig. 2(b,c)) situated at the extinction dip close to 1970 nm. In particular, the magnetic field (case 3 in Fig. 2(c)) at this wavelength is highly confined within the inner area of the trimer unit, which cannot be found at any of the other three wavelengths. This pattern has been intensively discussed in the literature and has been identified as a magnetic-based coil-type Fano resonance^23–25^. Here, the bright mode, formed by the three individual dipole modes being in electric resonance, couples to the magnetic resonance resulting in the circle-like polarization distribution. Therefore, destructive interference of the bright electric mode and the dark magnetic mode results in an extinction dip as confirmed in simulations and experiments. Several reports reveal that this magnetic Fano resonance requires additional symmetry breaking (modifying inter-particle gaps^22^ or geometry of trimer dots^23^, etc.) to be excited. This mode is assumed to be forbidden for an individual isolated symmetric trimer or for a sparse array of trimers^31,32^ since its total electric dipole moment is zero. In addition, its magnetic dipole moment is perpendicular to the sample surface. Therefore, this mode cannot be excited by a plane wave at normal incidence. However, in this closely packed trimer array where the coupling to neighbouring unit cells cannot be neglected, we experimentally observe spectral features for the magnetic Fano resonance modes. In the following sections, we will explore how the coupling between different trimer units can influence the overall optical response of the investigated arrays.

### Trimer plasmon modes in a square-shaped unit cell

**Figure 3:**
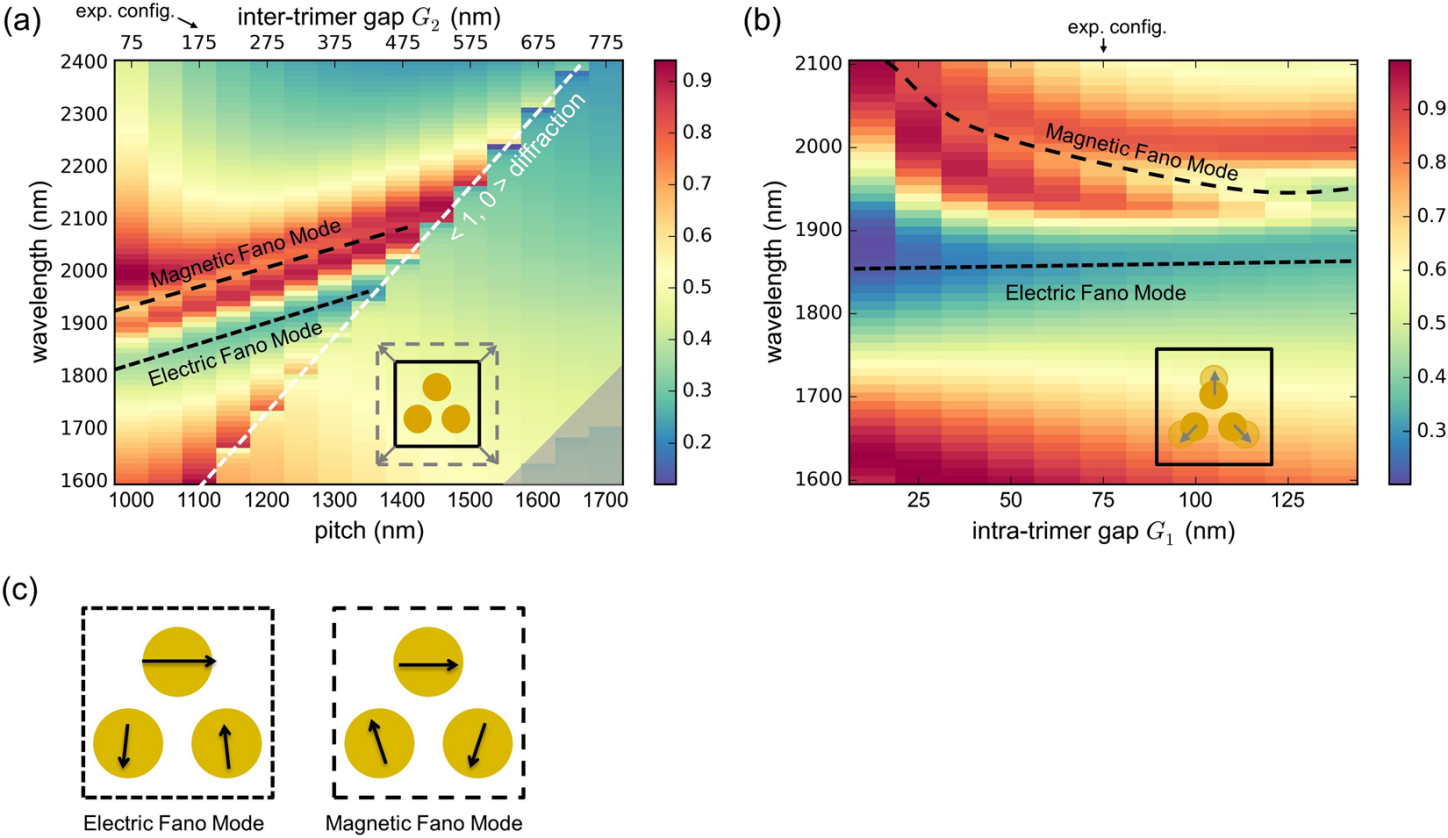
Simulations showing the spectral distribution of the extinction of one trimer in a square unit cell as a function of (**a**) pitch Λ (G_1_ = 75 nm) and (**b**) inter-trimer distance G_1_ (pitch is fixed to 1100 nm). The white dashed line in (**a**) indicates the onset of <1, 0> diffraction order in the index-matched configuration. The variation of the unit cell is schematically depicted in the lower right corner. The area denoting higher lattice modes is greyed out. The sketch shown in (**b**) displays the extinction in case the intra-trimer gaps are modified. (**c**) Schematics of the distribution of the trimer nanodot dipole moments relevant within the scope of this work. The evolutions of these two patterns in (**a**) and (**b**) are indicated by the appropriate dashed lines. The configurations targeted in the experiments presented here are indicated at the top of (**a**) and (**b**).

The following section discusses the impact of the intra-trimer gap (G_1_) and inter-timer gap (G_2_) on the spectral behaviour with the trimer located in the centre of a square-shaped unit cell. Here, the diameter (D) and height (H) of the dots is fixed to 425 nm and 40 nm, respectively. For convenience, we address our study to the periodic constant (pitch) Λ instead of the inter-timer gap G_2_ (Λ = 2  × D + G_1_ + G_2_). In detail, the spectral distribution of the extinction for various values of pitches (1000 nm < Λ < 1700 nm; steps of 50 nm; G_2_: 75 nm to 775 nm; G_1_ is fixed at 75 nm) is presented in Fig. 3(a). Additionally, Fig. 3(b) shows the simulated extinction spectra obtained by modifying the intra-trimer gap G_1_ from 12.5 to 132.5 nm in steps of 12.5 nm. Both calculations are carried out in the index-matched configuration (incident polarization is along the x-polarized direction) with the white dashed line (Fig. 3(a)) indicating the <1, 0> diffraction order in this configuration.

Overall, the extinction spectra can be dramatically modified by varying the inter- and intra-trimer gaps. In Fig. 3(a), electric and magnetric Fano resonance are observed at pitch values between 1000 and 1400 nm, which are indicated by short-dashed and long-dashed black lines. Within this range of pitches, the trimer-to-trimer coupling distance G_2_ is gradually varied while the dot-to-dot interaction remains the same (G_1_ is fixed). Noticeably, both patterns shift towards longer wavelengths, which indicates that the modal interactions can be modified by the lattice-induced coupling. Once the pitch exceeds 1400 nm, the extinctions only exhibit step-like lineshapes along the onset of the first diffraction order (λ = n × Λ) in the medium (n = 1.44), whereas the distributions referring to electric and magnetic Fano resonance can no longer be observed. More sophisticated lattice modes in the left corner are greyed out since they are beyond the discussion of this article.

Now we consider a complex coupling configuration where the intra-trimer gap G_1_ and inter-trimer gap G_2_ are both modified. Figure 3(b) displays simulated extinction spectra that are obtained by varying the intra-trimer gaps G_1_ (from to 12.5 nm to 137.5 nm) for a fixed pitch (Λ = 1100 nm). In this case, the gap size G_2_ is correspondingly changed from 237.5 to 112.5 nm. Surprisingly, the central spectral position of electric Fano resonance remains at approximately 1850 nm, while magnetic Fano resonance reduces in wavelength from approximately 2100 to 1970 nm. This blue-shift of the magnetic Fano resonance with increasing gap separation agrees well with the discussion in ref.^21^.

We need to point out that the emergence of the magnetic Fano resonance mode strongly relies on the near-field coupling of neighbouring unit cells. In Fig. 3(a), the spectral feature of the Fano resonance is most evident at a pitch of 1000 nm, where the inter-trimer gap distance reaches the minimum value investigated here (G_2_ = 75 nm, G_1_ is 75 nm). This amplitude enhancement for the Fano resonance mode is also visible in Fig. 3(b) when decreasing G_2_. In particular, at the minimum value of G_2_ (Fig. 3(b)), the array cannot be divided into individual trimer unit cells since the inter-trimer coupling of the lower two dots is stronger than the intra-trimer interactions, i.e., G_2_ is smaller than G_1_ (G_2_ 112.5 nm and G_1_ 137.5 nm).

### Trimer behaviour for the case when the coupling is varied along the horizontal or vertical coupling direction

**Figure 4:**
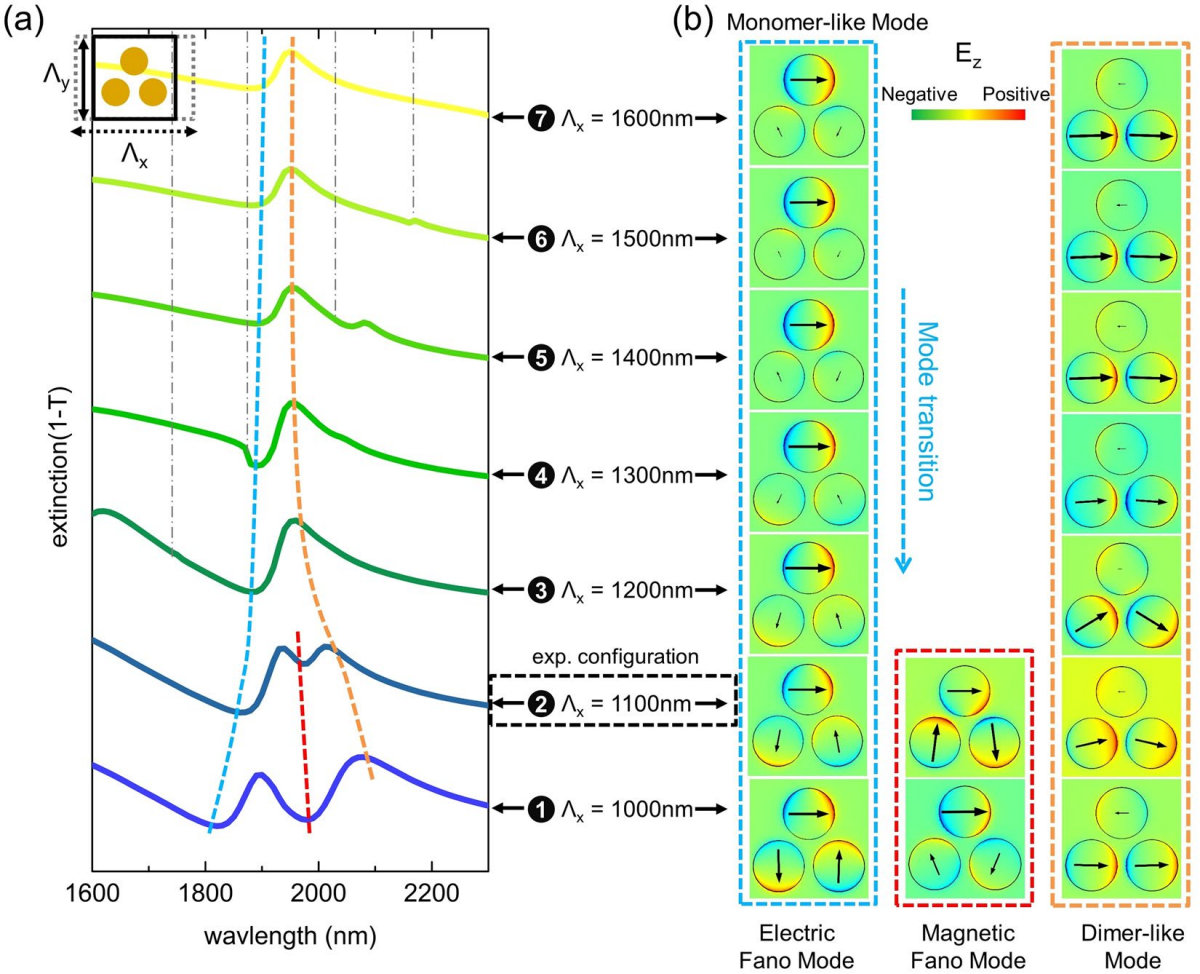
(**a**) Dependence of the spectral distribution of the extinction of the rectangular lattice trimer array for the case when the pitch is changed along the horizontal direction (x-axis) while no change is assumed along the vertical direction. The schematic in the top-left corner illustrates how the unit cell is modified. The vertical pitch is set to Λ_*y*_ = 1100 nm and Λ_*x*_ varies from 1000 to 1600 nm. (**b**) Corresponding simulated electric field patterns and polarization distributions at a fixed point in time at the wavelengths highlighted by the various dashed lines in (**a**). The designated number together with horizontal pitch Λ_*x*_ connects the spectral features and near-field patterns. Note, in the blue dashed frame, the transition of the mode from a monomer-like pattern to an electric Fano resonance pattern is clearly visible. The dashed coloured lines are guides-to-the-eye to highlight the spectral shift of the respective feature. The vertical dash-dot grey lines indicate the onsets of the <1, 0> diffraction orders. The experimental configuration measured is also highlighted by the box framed by the black dashed line.

**Figure 5:**
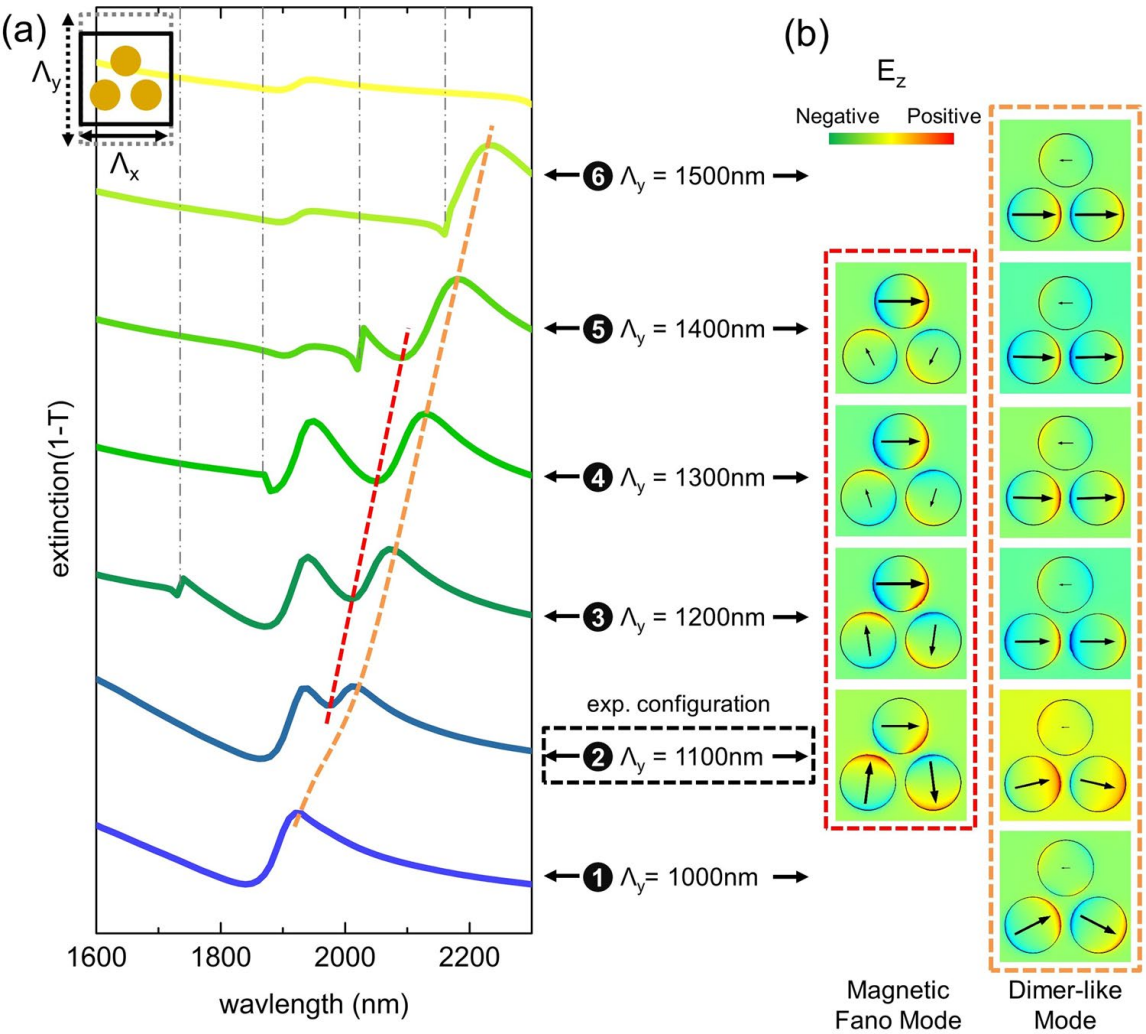
(**a**) Dependence of the spectral distribution of the extinction in case the pitch is changed along the vertical direction (y-axis, schematic in the top-left corner). Λ_*y*_ varies from 1000 to 1500 nm, while the horizontal pitch Λ_*x*_ is set to 1100 nm. (**b**) Corresponding simulated electric field patterns and polarization distribution at the wavelengths highlighted by the various dashed lines in (**a**). The designated number together with the horizontal pitch Λ_*y*_ connects spectral features and near-field patterns. The dashed coloured lines are guides-to-the-eye showing the spectral shift of the respective feature. The vertical dash-dot grey lines indicate the <0, 1> diffraction order. The experimentally investigated configuration is highlighted by the framed box.

In this section, we study the impact of the inter-unit cell coupling on the overall extinction spectra by altering the unit cell along the horizontal (x-axis) and vertical (y-axis) directions. For the following numerical simulations, arrays of trimers with a dot diameter of D = 425 nm, height of H = 40 nm and intra-trimer gap of G_1_ = 75 nm are numerically investigated assuming a homogenous RI environment. Using a periodic boundary condition, the corresponding simulation results are presented in Figs 4 and 5. Here, the horizontal and vertical edges of the rectangular unit cell are named in the following as Λ_*x*_ and Λ_*y*_, respectively.

We first examine the trimer spectra and mode profiles by merely extending Λ_*x*_ from 1000 to 1600 nm (G_2_: 75 to 675 nm) while keeping the vertical pitch constant (Λ_*y*_ = 1100 nm). The simulations are presented in Fig. 4 with a sketch used to show how the unit cell is modified (left upper corner in Fig. 4 (a)). Two distinct modes dominate the overall extinction response, namely, the monomer-like mode (extinction minima, blue-dashed line) and the dimer-like mode (extinction maxima, orange-dashed line), which both roughly remain at the same spectral position when Λ_*x*_ > 1200 nm (monomer mode: at approximately 1900 nm; dimer mode: close to 1950 nm). For such large horizontal pitches, the inter-trimer gaps G_2_ are larger than 275 nm, being nearly four times the size of the intra-trimer gap G_1_ (75 nm), with the result that the inter-trimer coupling is smaller than the inter-dot coupling. Hence, the trimers behave like individual elements and the interplay between the monomer mode and dimer mode is less pronounced.

As highlighted by the blue dashed line in Fig. 4(a), a transition from a monomer-liker mode (cases 7 to 3) to an electric Fano mode (case 2 and 1) is clearly observable. In particular, for Λ_*x*_ = 1000 nm, the inter-trimer coupling along the x-axis is maximal, with both electric and magnetic Fano modes clearly visible in the corresponding spectrum. Here, the magnetic Fano resonance mode becomes most prominent, i.e., the respective extinction minimum is most distinct among all the spectra presented.

The next step in the analysis targets the understanding of the impact of the vertical coupling on the optical response. Here, the extinction spectra for the trimer arrays in a rectangle cell with variation along the y-direction are calculated (G_1_ and G_2_ are fixed to 75 nm and 175 nm, respectively, with Λ_*x*_ = 1100 nm). Through varying Λ_*y*_, we obtain a set of extinction spectra that are shown in Fig. 5(a), which are accompanied by corresponding near-field distributions (Fig. 5(b)). Here, the trimer response becomes more complicated and highly asymmetric due to enhanced coupling between the trimers at the onset of the first diffraction order. Nevertheless, a dimer-like pattern (orange dashed line) can be observed for all six cases, while the corresponding extinction maximum shifts towards longer wavelengths when increasing Λ_*y*_. For cases 2 to 5 (pitch increases from 1100 to 1500 nm), the magnetic Fano resonance is formed, which is evidenced by the respective extinction minimum (red dashed curve in Fig. 5(a)). Inspection of the corresponding near field patterns (red frame) shows that for the case when Λ_*y*_ is increased, the amplitude of the excitation of the lower two dots is reduced while the top dot shows a gradually stronger response. Once Λ_*y*_ > 1500 nm, the magnetic Fano resonance cannot be observed anymore.

At this stage, we would like to conclude the discussion of the impact of the near-field coupling on the formation of the magnetic Fano resonance obtained from the simulated results presented in Figs 3, 4 and 5. First, the spatial distribution of the trimers within the array matters. We clearly observe that in the experiments and in simulations, a magnetic Fano resonance mode is formed in densely packed arrays of symmetrical trimers, which should be forbidden for an isolated symmetric trimer based on group theory^22,32,34^. Here, we attribute the formation of the magnetic Fano resonance to the symmetry breaking imposed by the square lattice, i.e., to the fact that the trimers and the array have different symmetries. Specifically, the square-shaped lattice breaks the three-fold symmetry of individual trimers, enabling the magnetic and electric modes of the trimer to interact. Second, symmetry breaking-mediated formation of the magnetic Fano resonance only works under particular conditions, i.e., only for certain combinations of lattice parameters, wavelength and incident light polarization. For instance, according to the square-shaped unit cell studied in Fig. 3(a), the pitch should not exceed 1400 nm to trigger the formation of the magnetic Fano resonance. For this configuration, the impact of diffraction can be suppressed below the actual operation wavelength while the inter-unit cell coupling remains effective. Finally, we would like to emphasize again that the trimer-to-timer coupling plays a more important role for the formation of the magnetic Fano resonance compared to the dot-to-dot interaction within a trimer unit. This phenomenon can be found in Figs 3(b) and 4(a), where a complete loop-like polarization is gradually formed when decreasing the inter-trimer gap G_2_.

### Trimer plasmon modes in various RI environment

**Figure 6:**
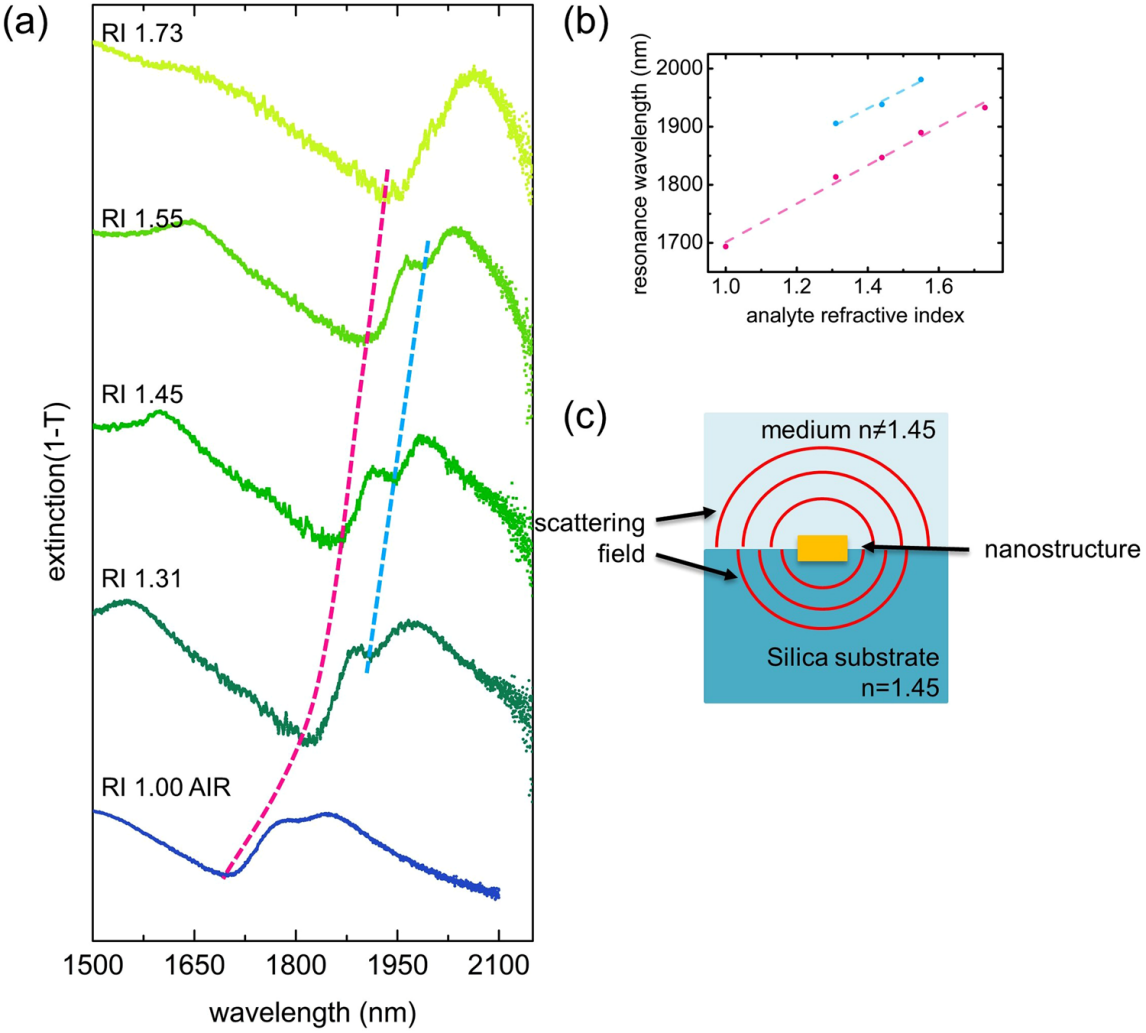
(**a**) Measured extinction spectra for the trimer array for different vertical RI configurations (the superstrate refractive index was changed by immersing the sample into different liquids). The dashed magenta and blue curves are guides-to-the-eye and indicate the spectral redshift of the electric and magnetic Fano resonances(shown in Fig. 2), respectively. (**b**) Resonance wavelength (left axis) as a function of superstrate RIs. The dashed lines are linear fits to the data points. (**c**) Illustration showing the different scattering fields in the situation of an inhomogeneous refractive index environment.

Detecting small changes of the refractive index in nanoscale environments is of key importance for numerous fields particularly within the fields of bioanalytics and molecular sensing, representing a topic that is intensively addressed by the nanophotonics community^35–39^. To understand the impact of the RI environment on the optical response of the trimer array and to determine the related RI sensitivity, we immersed the samples into different RI liquids (n = 1.31 to 1.73) and measured the extinction under normal incidence (Fig. 6). In general, the resonance dip, being located near 1650 nm (electric Fano resonance in Fig. 2) in the situation of an air superstrate, shifts towards longer wavelengths as the RI of the liquids increases (this spectral red-shift is highlighted in Fig. 6(a) by the dashed magenta line). The magnetic Fano resonance mode is only found in three of the five extinction spectra (n = 1.31, 1.44 and 1.55). The corresponding wavelengths for the resonances are plotted in Fig. 6(b) and are fitted by linear functions to obtain the RI sensitivity. This procedure yields an RI sensitivity of 312.8 nm/RIU (coefficient of determination *R*^2^ = 96.9%) for the electric Fano-like mode and 330.5 nm/RIU (coefficient of determination *R*^2^ = 98.8%) for the magnetic Fano resonance mode. Compared to sensitivities obtained in other types of plasmonic structures (nanodisks (∼180 nm/RIU in VIS^40^), lattices of trimers (∼170 nm/RIU in VIS^41^, ∼370 nm/RIU in IR^41^)), our densely packed trimer arrays offer sensitivities of the same order, which are comparable to other Fano resonance sensors (approximately 300 nm/RIU in nanohole quadrumers^42^ and heptamer cluster^43^). One advantage of our structure is that the area covered by the array can be minimized to several μm^2^ due to the dense configuration. As reported in ref.^44^, we designed a trimer-based fibre sensor, where the diameter of the sensing area is approximately 100 μm^2^ enabling one to perform sensing with the light from the fibre core only, which would otherwise be difficult if the array was very sparse.

It is important to note that the magnetic Fano resonance mode is more pronounced for the case when the particle array is immersed in the liquid with an RI equal to that of the substrate. In this case, a unique relationship between the scattered field and the pitch is obtained because the wavenumber of the scattered wave is the same as that in the substrate and in the superstrate. For the RI-mismatched situation (the bottom and top case in Fig. 6(a)), the two different RIs impose two scattered waves at the same vacuum wavelength (schematically illustrated in Fig. 6(c)). Hence, the distribution of the scattered energy into two waves with different wavenumbers leads to weaker spectral features rather than the strong feature realized in the case of a homogeneous environment.

## Conclusion

Artificial nanostructures have appreciably promoted the development of nanophotonics research in the last decade, with a multitude of novel optical effects discovered with one of the most important examples being the Fano resonance. In this report, we have studied the impact of the lattice geometry on the optical response of nanotrimers arranged in densely packed square arrays at infrared wavelengths. We found that in experiments as well as in simulations, electric and magnetic Fano resonance modes can be formed in cases when the inter-trimer distances are sufficiently small, i.e., arise solely due to the array arrangement. In particular, the observation that magnetic Fano resonances can be excited in a system that consists of a symmetric nanostructure unit supporting a magnetic Fano resonance as a dark mode only clearly shows that the lattice arrangement imposes a symmetry breaking, making the magnetic Fano resonance visible in the spectral response of the array. By varying the array parameters, we clearly reveal a transition from an isolated trimer mode into a regime of strong near-field coupling, showing magnetic Fano resonances with loop-type magnetic field distributions. This otherwise hard-to-observe excitation only emerges due to the presence of the neighbouring trimer units and the symmetry mismatch between the square lattice and three-fold symmetry of the nanotrimers. Finally, we reveal the impact of different refractive index environments on the optical response of the trimer arrays, with the sensitivity measured to be approximately 300 nm/RIU.

By transferring the concept of lattice-induced symmetry breaking to other nanostructures, our findings provide a pathway towards observing novel kinds of optical excitations and yield a new degree of freedom to design artificial nanocomponents and -devices. Promising applications for the trimer lattice can be envisioned within fields such as nonlinear optics to generate frequencies at desired wavelengths or bioanalytics via high precision sensing^44^. Both examples exploit the unique features of the strongly enhanced electric and magnetic fields generated within the centre of the structure.

## Supplementary information

Supplementary_Info
