# Supplementary material for: Symmetry-breaking induced magnetic Fano resonances in densely packed arrays of symmetric nanotrimers
Source: Sci Rep. 2019 Feb 27;9:2873. doi: 10.1038/s41598-019-39779-x (PMC6393417; doi:10.1038/s41598-019-39779-x)
Supplement: Supplementary file 1 — Supplementary_Info [file 41598_2019_39779_MOESM1_ESM.docx]

Supplementary Information

Symmetry-breaking induced magnetic Fano resonances in densely packed arrays of symmetric nanotrimers

Ning Wang,^1^ Matthias Zeisberger,^1^ Uwe Huebner,^1^ Vincenzo Giannini,^2,3^ and Markus A. Schmidt ^1, 4, 5 *^

1 Leibniz Institute of Photonic Technology, Albert-Einstein-Str. 9, 07745 Jena, Germany

2 The Blackett Laboratory, Department of Physics, Imperial College London, London SW7 2AZ, UK

3 Instituto de Estructura de la Materia (IEM-CSIC), Consejo Superior de Investigaciones Científicas, Madrid 28006, Spain

4 Abbe School of Photonics and Faculty of Physics, Max-Wien-Platz 1, 07743 Jena, Germany

5 Otto Schott Institute of Materials Research, Fraunhoferstr. 6, 07743 Jena, Germany

*E-mail: [markus-alexander.schmidt@uni-jena.de](mailto:markus-alexander.schmidt@uni-jena.de)

### Section 1: Characterization of trimers with increasing dot diameter

**Figure S1.**Transmission measurements (A) and simulations (C) of the symmetric trimer geometry. Measured spectral distribution of the transmission of various symmetric trimers, with the corresponding unit cell shown by the SEM images between the two plots ((B), the scale bar is 500 nm). The polarization of incident light is parallel to the axis of bottom two dots (i.e., is along the x-axis as indicated by the double headed arrow on top of the SEM images).


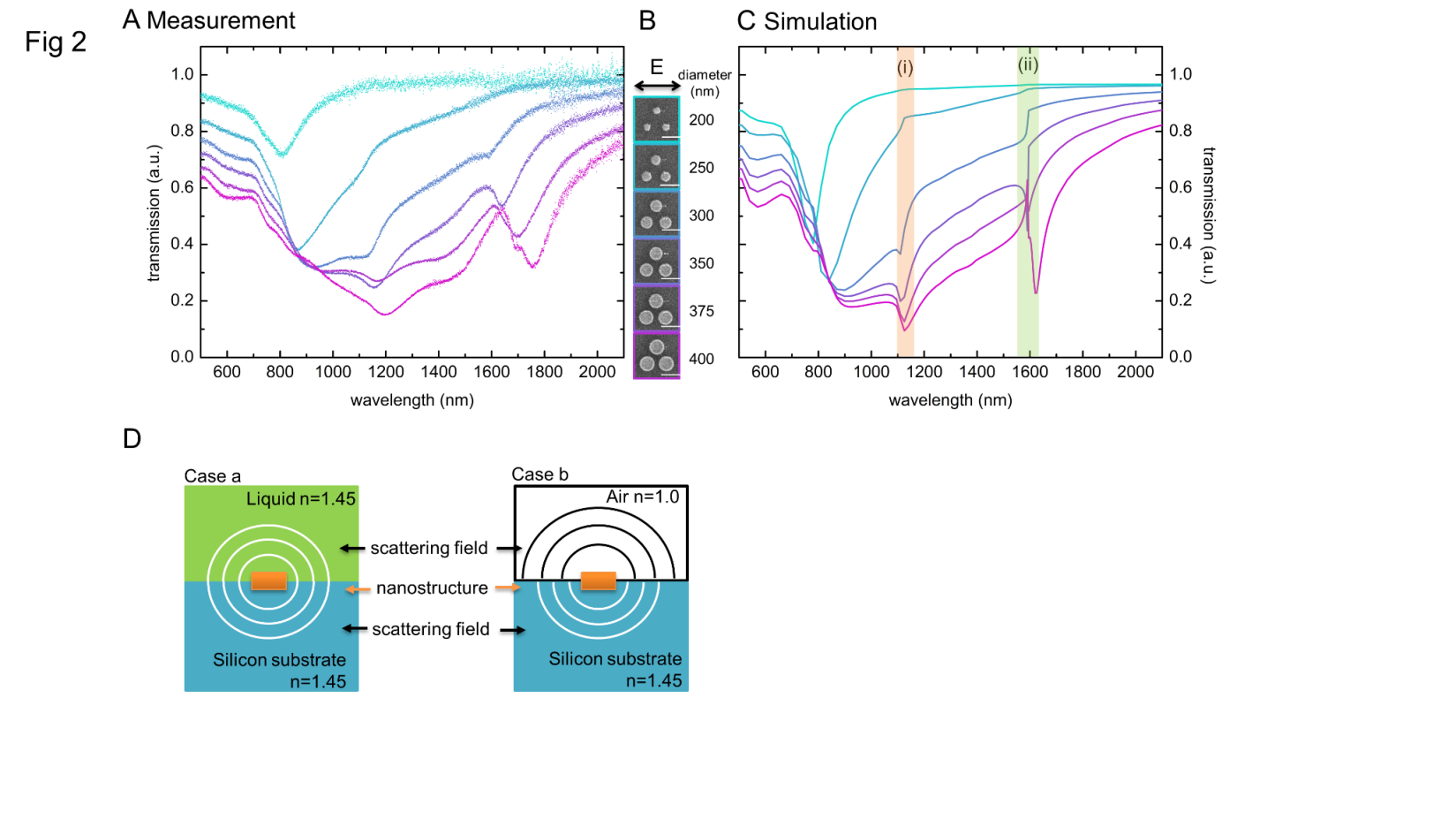


The spectral distribution of the transmission of different types of symmetric trimers is measured polarization-resolved and compared to numerical simulations. In figure S1, trimers arrays with gradually increasing inter- and intra-trimer coupling, mediated by decreasing the interparticle edge-to-edge distance have been investigated. Specifically, the diameter of trimer dots increases from 200 nm, 250 nm, 300 nm, 350 nm, 375 nm to 400 nm respectively, whereas the center-to-center distance between two dots is fixed to 500 nm. As the diameter of dots increases, the gap G_1_ and the gap G_2_ reduce to 300 nm, 250 nm, 200 nm, 150 nm, 125 nm, 100 nm and 400 nm, 350 nm, 250 nm, 225 nm, 200 nm respectively. In short, the trimer units transit from isolated particles to a system of coupled nanodisks in case the inter-particle distances are reduced. The measured transmission spectra of the symmetric trimer in x-polarization are shown in figure S1 (A) with corresponding SEM images (right column indicating the dot diameter) shown in Figure S1 (B). Each configuration shows that resonance dips shift to longer wavelength for increasing nanodisk diameters because of the well-known relation between the LSPR dip position and the geometry of the nanoparticle. However, the transmission lineshape of the trimers changes substantially and becomes increasingly asymmetric for larger disk sizes. For the first two situations (diameter 200 nm and 250 nm), only one pronounced dip near 800 nm is observed. While in the remaining four cases (diameter from 300 nm to 400 nm), two other dips appear close to 1100 nm and 1700 nm and a sharp transmission maximum emerges at around 1600 nm.

Section 2: Characterization of the polarization dependence of the trimer arrays

In the following section, the optical properties of the trimers (dimeter 400 nm) are experimentally analyzed by rotating the incident light polarization from 0° to 90°. The corresponding transmission spectra from 1400 nm to 2000 nm are shown in figure S2 (A). The polarization angle is defined relative to the center connection line between the lower two dots as indicated in figure S2 (B). In detail, the trimers are illuminated under the normal incident light with polarization angles of 0°, 15°, 30°, 45°, 60°, 75° and 90° respectively.


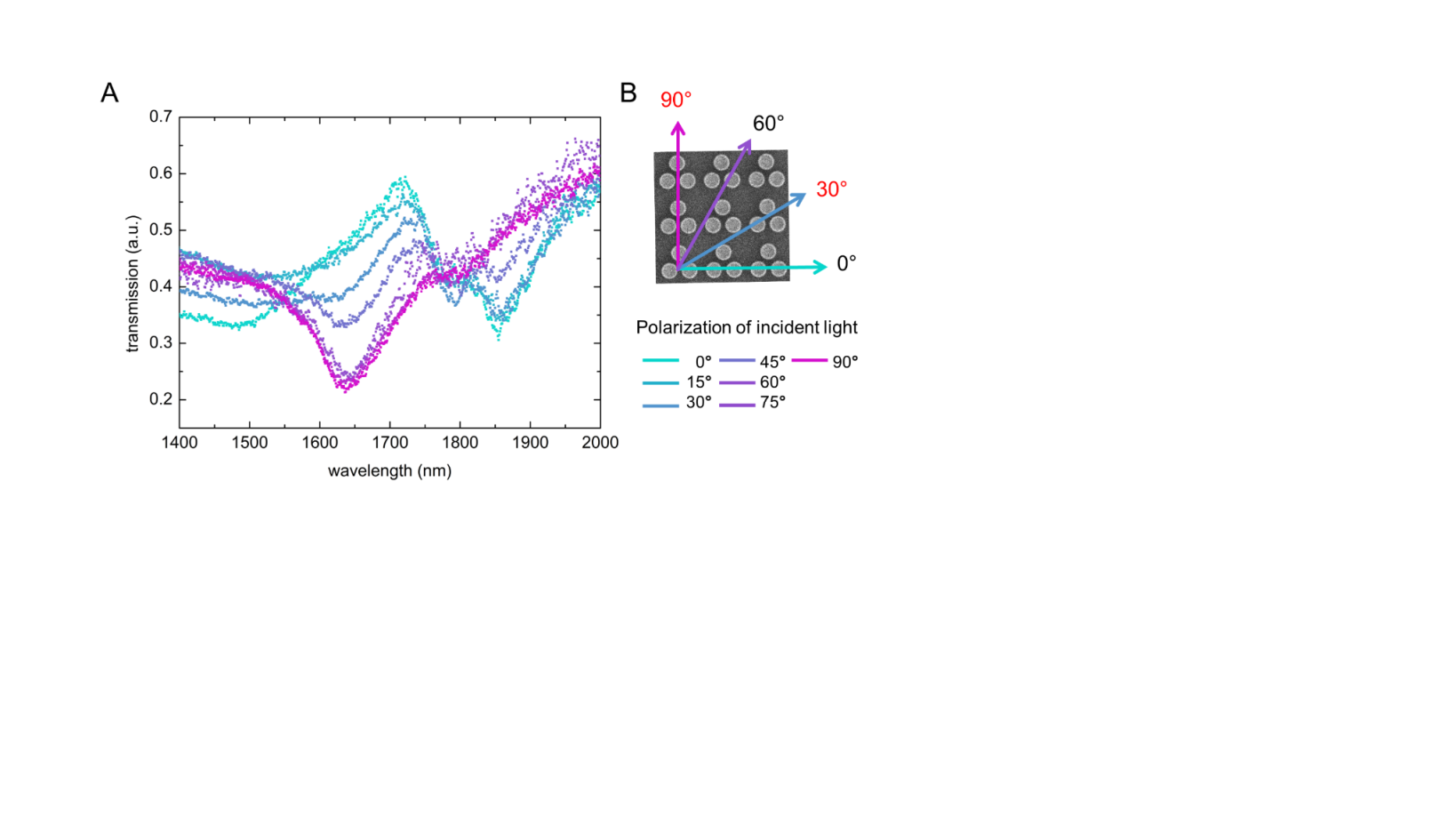


Figure S2. (A) Transmission spectra of the trimer array with nanodisk diameter of 400 nm for different angles of input polarization (from 0° to 90° in step of 15°). Remarkably at around 1650 nm, the peak gradually changes to a pronounced dip. (B) Illustration of different polarization directions with respect to the trimer array geometry.

In contrast to other works where the light polarization does not have a strong influence on the optical property of an isolated trimer, the spectral distributions of the transmission of the close-packed trimer array dramatically depend on the incident light polarization.

Section 3: Fabrication process.

The fabrication of the trimer arrays starts with a pre-cleaned fused-silica-chip (10×10 mm², 1 mm thick). The pre-cleaning is done using Caro’s acid (Peroxymonosulfuric acid) and oxygen-plasma cleaning. First, the chip is vapor deposited with 3 nm titanium as an adhesive layer and 40 nm gold (Roth&Rau “Microsys 600”). Then the gold surface is coated with a 130-nm thick negative-tone e-beam resist ma-N 2401 (micro resist technology GmbH, spin-process, tempered 3 min at 90°C on a hotplate). The electron beam lithography is performed using the e-beam tool Vistec 350OS (50 keV). This shaped beam system is equipped with the Character Projection technique which allowed the fast-quasi-parallel exposure of nanopatterns on large areas. Here, the trimer patterns are exposed by using the “pinhole-microreticle” with a diameter 350 nm. The structure size is fine-controlled by the electron dose in a range from 330 to 420 nm. Arrays of nanodisk-trimers with different plate sizes are arranged on the chip to have variations for the optical experiments. The development of the resist is done 30 secs in AZ MIF-726 developer and rinse by water. The trimer-arrays are etched into the gold-film by using Ar+-ion beam etching (IBE, tool: “Multiplas II” from 4-TEC). After the IBE, the residues are removed wet-chemically (rinsed in an ultrasonic acetone bath and isopropanol), followed by oxygen plasma cleaning (50 W, 1 min). At the end, a 10-nm alumina film is deposited on the entire chip surface using an atomic layer deposition process (plasma enhanced ALD, tool “Oxford Opal”). This alumina film serves as a mechanical surface protection layer in the following optical near-field measurements as well as transmission measurements.

Section 4: Transmission setup.

Figure S3 shows the transmission measurement setup. The sample is placed on the two-axis stage with normal incident light from a supercontinuum laser (NKT Photonics SuperK Compact) covering a wavelength range from the visible to the infrared regime. A polarizer is used to control the light polarization. The lower beam splitter is used to couple imaging light working at 660 nm to the sample. During experiments, this beam splitter was removed. Two 20 times objectives are employed to obtain a focused beam with a spot diameter around 10 µm which is much smaller than the size of trimer arrays. The transmitted light spectrum is recorded by the optical spectrum analyzer (Instrument Systems SP320-124). Therefore, the transmission curve is acquired by the power ratio of two spectra with one beam on the trimers and the other beam passing through a plane silica substrate. With the additional illumination and a visible camera, the sample and the beam position can be observed.


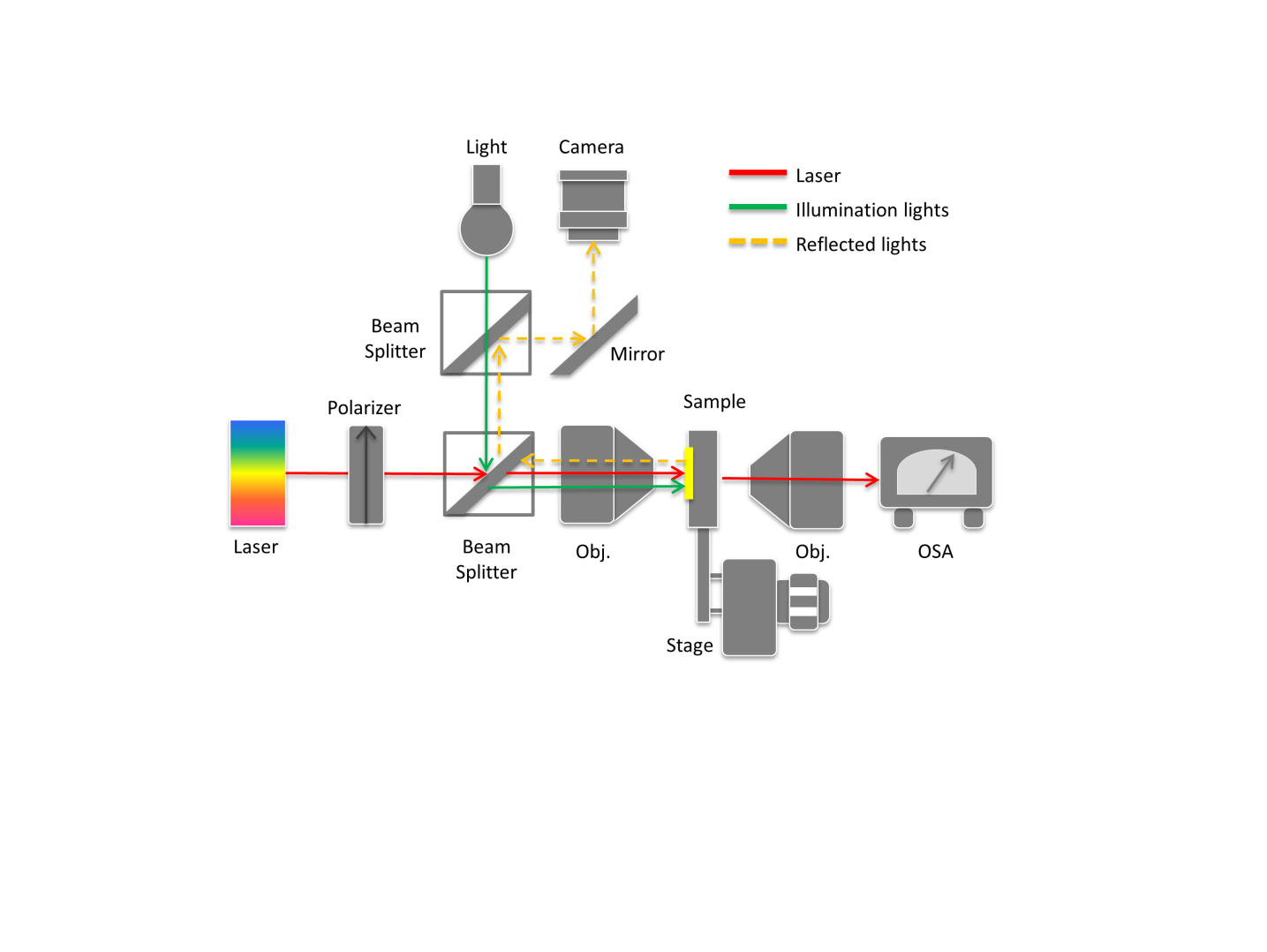


Figure S3. Experiment setup for transmission spectrum measurement.

Section 5: The 3D simulation model.

The simulations are carried out by Comsol Multiphysics with the model geometry shown in Figure S4. In all simulations, the structure consisted of three identical disks arranged on the surface of the substrate. The medium above the trimer can be varied in its refractive index. For most of the investigations presented in the main paper the refractive index is the same as that of the substrate. For the simulations shown in Fig. S1 c) the upper medium was air. The heights of air and substrate are both 500 nm and the height of dots is fixed at 40 nm. In order to resemble the real sample situation as much as possible, periodical boundary conditions are applied. Therefore, the periodic ports consisting of a bottom excitation port and a top output port, are employed to obtain transmission curve according to the zero-th diffraction order which is the relevant part of the transmission that is detected in the experiment (The higher diffraction orders do not propagate along the axis and will not reach the detector). The permittivity of gold is taken from the experimental data shown in of Ref 1. We fix the permittivity of air to unity and fix the substrate permittivity to 2.09 since there is only a slight variation of the refractive index in the measured spectrum range.

To reach sufficiently high simulation accuracy the dots need to be finely meshed. Here the maximum mesh value is 5 nm. The parameter sweep module in this study is used for the spectrum simulation covering a range from 1400 nm to 2100 nm. In this the specific sample shown in Figure S4, a symmetric trimer with a diameter of 400 nm, an intra-trimer gap of 100 nm and the pitch of 1000 nm is simulated. Here the electromagnetic field distribution at a wavelength of 1480 nm shows the lower two dots resonate in oppose directions.


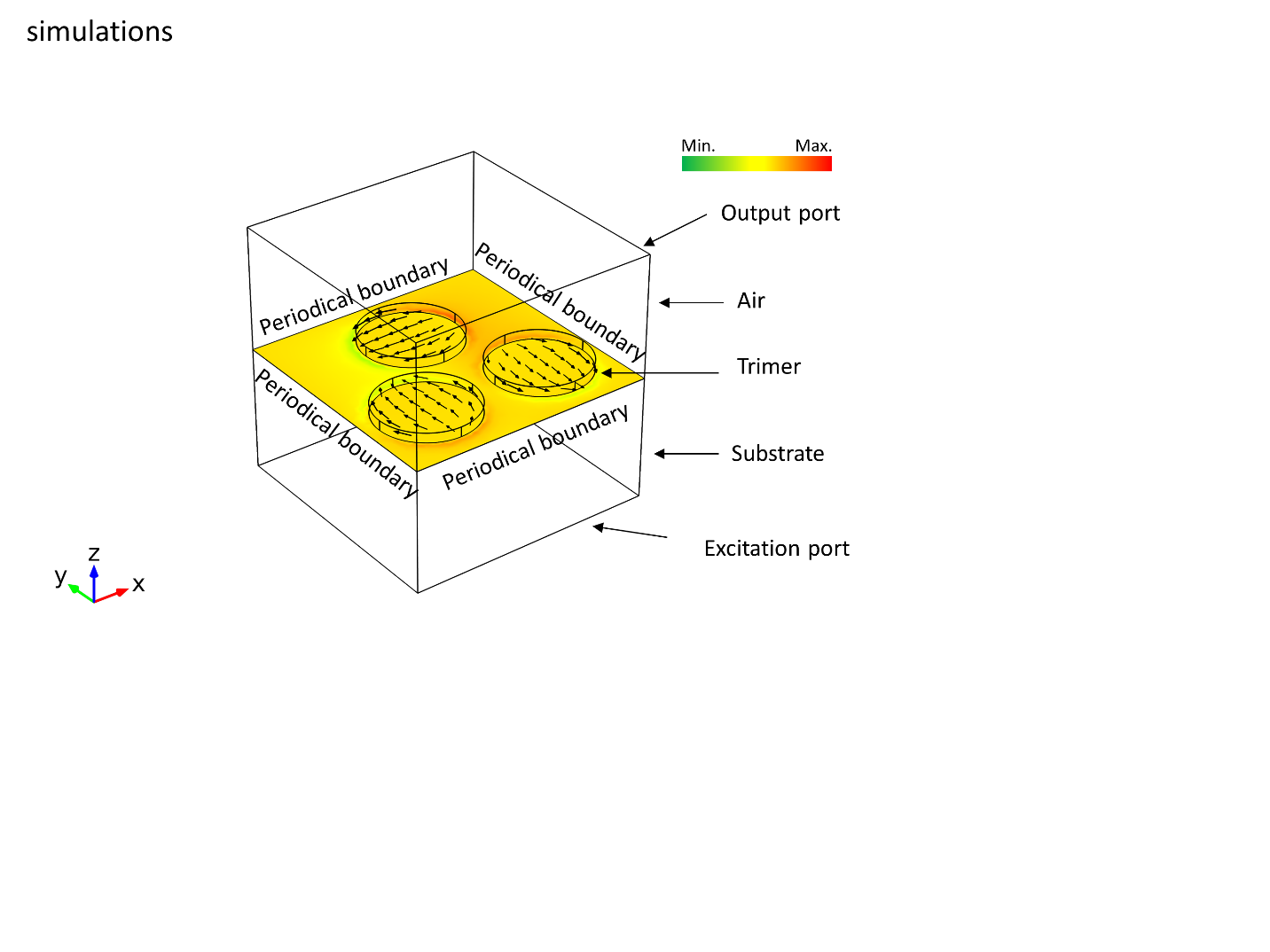


Figure S4. Simulation model. The RF module in Comsol Multiphysics is employed for 3D nanostructure simulation.

Section 6: Trimer hybridization diagram


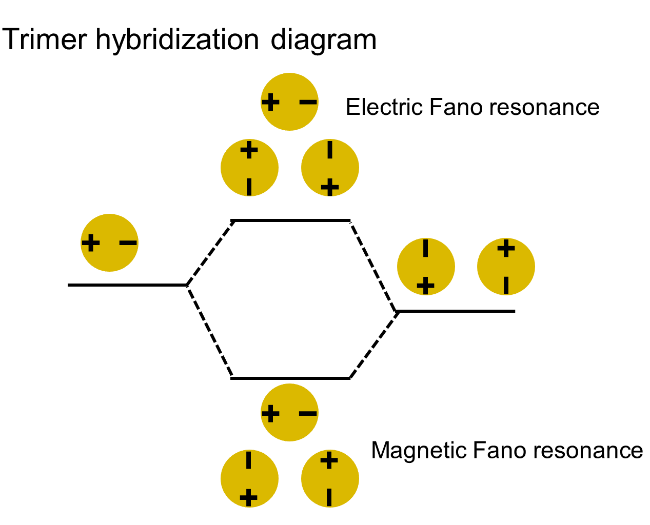


The trimer resonance can be understood as hybridization of a dipole mode with a quadrupole mode^[2,3]^. As shown in Fig. S5, a dipole resonance, supported by the upper dot, interferes with a quadrupole-like resonance of the two lower dots. Note that the quadrupole mode is often referred as dark mode, which can be triggered by near-field coupling instead of external electromagnetic field. The hybridization of the two modes yields two different charge distributions resembling electric Fano-like (top case) and magnetic Fano-like modes (bottom case).

Reference:

1 Rakić, A. D., Djurišić, A. B., Elazar, J. M., & Majewski, M. L. (1998). Optical properties of metallic films for vertical-cavity optoelectronic devices. *Applied optics, 37*(22), 5271-5283.

2 Abasahl, B., Santschi, C., & Martin, O. J. (2014). Quantitative extraction of equivalent lumped circuit elements for complex plasmonic nanostructures. *ACS Photonics*, *1*(5), 403-407.

3 Prodan, E., Radloff, C., Halas, N. J., & Nordlander, P. (2003). A hybridization model for the plasmon response of complex nanostructures. *science*, *302*(5644), 419-422.
